# Proceedings of the 22nd International Cancer Imaging Society Meeting and Annual Teaching Course

**DOI:** 10.1186/s40644-023-00611-5

**Published:** 2023-10-12

## Oral presentations

### O1 Performance of deep learning model on automated lesion detection: virtual monoenergetic images on dual-energy CT

#### Yin-Tzu Lin, Yenpo Lin, Kueian Chen, Gigin Lin

##### Linkou Chang Gung Memorial Hospital, Taoyuan, Taiwan

###### **Correspondence:** Yin-Tzu Lin (lin.ytzu@gmail.com)

*Cancer Imaging 2023,* **23(1):**O1

**Aim**

We aimed to evaluate the performance of a deep learning (DL) model for automated lesion detection on virtual monoenergetic images (VMI) from dual-energy CT (DECT).

**Materials and Methods**

Lesion detection using a DL model was performed on axial contrast-enhanced CT images of chest and abdominopelvis obtained in 5 mm slice thickness and different energy level from 40~140 keV with 10-keV intervals, for gynecological cancer evaluation. The lesion-based analysis was compared based on different energies (the 40 keV, 50 keV, 60 keV and 70 keV as low energy VMI) and in different groups according to lesion location (chest versus abdominopelvis) and lesion size (≥ 1 cm as measurable versus < 1 cm as non-measurable).

**Results**

The final analysis comprised 257 lesions, including 156 measurable and 101 non-measurable lesions. In the measurable group, the DL model detected up to 81.4% of lesions. Using low energy VMI, the DL model completely detected all the measurable lesions that were detected via all energy levels. The DL model with low energy VMI had a better true positive rate (80.8%) as compared with higher energy VMI (*P* < .05), with a better true positive rate in the chest (88.6%) than the abdominopelvis (80.2%, *P* < .05). The DL model detected only 44.6% of the non-measurable lesions, with the highest precision in 100 keV.

**Conclusion**

Deep learning model using lower energy levels on DECT may enhance performance for automated measurable lesion detection, with a better true positive rate in the chest than the abdominopelvis.

### O2 Patterns of pseudoprogression across different cancer entities treated with immune checkpoint inhibitors

#### Wolfgang Kunz

##### LMU Munich, Munich, Germany

###### **Correspondence:** Wolfgang Kunz (wolfgang.kunz@med.lmu.de)

*Cancer Imaging 2023,* **23(1):**O2

**Aim**

Pseudoprogression (PsPD) is a rare response pattern to immune checkpoint inhibitor (ICI) therapy in oncology. This study aims to reveal imaging features of PsPD, and their association to other relevant findings.

**Materials and Methods**

Patients with PsPD who had at least three consecutive cross-sectional imaging studies at our comprehensive cancer centre were retrospectively analyzed. Treatment response was assessed according to immune Response Evaluation Criteria in Solid Tumours (iRECIST). PsPD was defined as the occurrence of immune unconfirmed progressive disease (iUPD) without follow-up confirmation. Target lesions (TL), non-target lesions (NTL), new lesions (NL) were analyzed over time.

**Results**

Thirty-two patients were included (mean age: 66.7 ± 13.6 years, 21.9 % female) with mean baseline STL of 69.7 mm ± 55.6 mm. PsPD was observed in twenty-six patients (81.3 %) at FU1, and no cases occurred after FU4. Patients with iUPD exhibited the following: TL increase in twelve patients, (37.5 %), NTL increase in seven patients (21.9 %), NL appearance in six patients (18.8 %), and combinations thereof in four patients (12.5 %). The mean and maximum increase for first iUPD in sum of TL was 19.8 mm and 96.8 mm (+700.8 %). The mean and maximum decrease in sum of TL between iUPD and consecutive follow-up was -19.1 mm and -114.8 mm (-60.9 %) respectively.

**Conclusion**

PsPD occurred most frequently at FU1 after initiation of ICI treatment. The two most prevalent reasons for PsPD were TL und NTL progression, with an increase in TL diameter commonly below +100 %. These findings may guide decision-making of ICI continuation in suspected PsPD.

### O3 Clinical utility of deep learning algorithm to achieve pin-sharp images quicker: real world experience across multiple sites

#### Peter Strouhal^1^, James Berry^1^, Edgar Mutemererwa^2^, Jane Hickey^3^, Stephen Hindle^3^, Stefano Persichini^3^, David Dooley^4^, Niamh Brennan^5^

##### ^1^Alliance Medical, Warwick, United Kingdom; ^2^Alliance Medical, Sidcup, United Kingdom; ^3^GE HealthCare, Chalfont St Giles, United Kingdom; ^4^Alliance Medical, Galway, Ireland; ^5^Alliance Medical, Galway, United Kingdom

###### **Correspondence:** Peter Strouhal (pstrouhal@alliance.co.uk)

*Cancer Imaging 2023,* **23(1):**O3

**Background**

AIR™ Recon DL is a pioneering deep-learning-based reconstruction algorithm, enabling modern MRI scanners to achieve pin-sharp images quicker, by removing noise and ringing from raw images. Improved signal-to-noise allows scan time reduction up to 50%, smoothing workflow and enhancing patient experience. Alliance Medical UK and Alliance Medical Ireland worked collaboratively with GE HealthCare and two local hospital partners to introduce this technology into routine MRI service.

**Purpose**

Provide overview of the adoption process of this new AI technology within two different general MRI units, to show the real-world benefits in scanner image quality and patient throughput.

**Learning Outcomes**

- Explain Air Recon DL reconstructions available.
- Discuss how these bring benefit to image data sets; and discuss any limitations.
- Explain the challenges experienced and how they might be mitigated.

**Application to Practice**

- Demonstrate realistic and achievable approach to implementing these reconstructions into routine clinical use.
- Evidence the benefits found at two difference scanning facilities, to show the applicability in real-world settings.
- Highlight the challenges experienced and discuss their impact on current / future practice.

**Summary of Content**

An overview of the implementation and adoption of Air Recon DL MRI reconstructions will be presented; showing how this enhances image quality and simultaneously reduces scan times of routine MRI sequences to support safe, effective and quality patient pathways. As well as a review of the successes, challenges and lessons learnt will be shared.

### O4 Applications of spectral CT technology in routine out-patient/CDC setting

#### Peter Strouhal^1^, Heathcote Ann^1^, Burn Paul^2^, Dominic Kite^2^, Jane Robinson^3^, Jeevan Gunararatnam^3^

##### ^1^Alliance Medical, Warwick, United Kingdom; ^2^Somerset NHS FT, Taunton, United Kingdom; ^3^Philips, Farnborough, United Kingdom

###### **Correspondence:** Peter Strouhal (pstrouhal@alliance.co.uk)

*Cancer Imaging 2023,* **23(1):**O4

**Background**

Philips IQ on Spectral CT uses single source in conjunction with dual layer detector: so-called detector-based technology; simultaneously absorbing and differentiating high and low energy acquired within single scan without any special mode. Thus, every examination has spectral information available even if not considered as a requirement prior to scanning. Alliance Medical worked collaboratively with Philips and local hospital partner/Community Diagnostic Centre to introduce spectral technology into routine clinical practice.

**Purpose**

This work illustrates the initial findings of Spectral technology, low mono-energetic (Low MonoE) and virtual non-contrast (VNC) reconstructions, to aid image evaluation in clinical practice in Taunton Community Diagnostic Centre.

**Learning Outcomes**

- Explain spectral reconstructions available.
- Discuss why spectral reconstructions were added to image sets.
- Explain challenges experienced and how they were mitigated.

**Application to Practice**

- Demonstrate realistic, achievable approach to adopting spectral reconstructions into routine clinical practice, specifically prostate cancer CT.
- Evidence benefits of Low MonoE and VNC reconstructions to specific datasets.
- Explain why not applied to all image sets.
- Highlight challenges experienced and discuss their impact on current / future practice.

**Summary of Content**

An overview of implementing and adopting spectral reconstructions will be presented; showing benefits over standard CT data to support a safe, effective and quality patient pathway. As well as a review of the successes, challenges and lessons learnt will be shared.

### O5 Feasibility of AI as a liver screening tool for staging CT scans of colorectal cancer patients

#### Usman Bashir^1^, Greg Slabaugh^2^, Qianni Zhang^2^

##### ^1^Barts NHS Trust, London, United Kingdom; ^2^Queen Mary University, London, United Kingdom

###### **Correspondence:** Usman Bashir (drusmanbashir@gmail.com)

*Cancer Imaging 2023,* **23(1):**O5

**Aims**

We aimed to measure the detection rate of a modern deep-learning architecture (nnU-Net) for liver metastases in colorectal cancer patients undergoing staging CT.

**Methods**

An ensemble-network of five nnU-Net models was trained on 230 CT scans with liver metastases annotated by a radiologist. The training set included 30 cases specifically containing sub-centimetre lesions retrieved from a text-based search of our hospital records. A separate test-set comprising 30 normal and 30 abnormal CT scans was used for reporting.

**Results**

The 30 abnormal CT scans contained 353 lesions (median 9 lesions per-scan; range 3-51). Of these, 190 lesions measured <10mm, 115 lesions measured 10-20mm, and 48 lesions measured >20mm. nnU-Net detected 80.1% lesions overall, and 72.1%, 85.2%, and 100% respectively from the three size-categories. On per-patient analysis, all patients in the metastasis group (*n* = 30) were correctly classified as having metastases. Of the normal CT scans (*n* = 30), 24 were correctly classified as normal whereas six scans were misclassified as having lesions (median 1 lesion per-scan; range 1-4). Per-patient sensitivity, specificity, positive predictive value, negative predictive value, and accuracy were 100%, 80%, 83.3%, 100%, and 90% respectively.

**Conclusion**

nn-UNet has potential to serve as a computer-aided screening tool for colorectal liver cancer metastases owing to its high sensitivity and negative predictive value.

### O6 Lugano criteria modification by pre-infusion kinetics improves survival prediction in chimeric antigen receptor T-cell therapy for lymphoma

#### Wolfgang Kunz, Michael Winkelmann

##### LMU Munich, Munich, Germany

###### **Correspondence:** Wolfgang Kunz (wolfgang.kunz@med.lmu.de)

*Cancer Imaging 2023,* **23(1):**O6

**Aim**

Chimeric antigen receptor T-cell therapy (CART) is effective for patients with refractory or relapsed lymphoma. We aimed to improve the prediction of Lugano criteria for overall survival (OS) by including the pre-infusion tumour growth rate (TGR^pre-BL^) and its change to 30-day follow-up (FU) (TGR^post-BL^).

**Materials & Methods**

Consecutive patients with pre-baseline (pre-BL), baseline (BL) and 30-day FU imaging before CART were included. TGR was defined as change of Lugano-based tumour burden between pre-BL, BL and FU examinations per time. Response was determined using Lugano criteria. Proportional Cox regression analysis studied association of TGR with OS.

**Results**

59 patients met the inclusion criteria. At 30-day FU, 16% had CR, 42% PR, 25% SD, and 19% PD. The Median TGR^pre-BL^ was -0.6 mm^2^/d, 24.4 mm^2^/d, -5.1 mm^2^/d, and 18.6 mm^2^/d and the median TGR^post-BL^ was -16.7 mm^2^/d, -102.0 mm^2^/d, -19.8 mm^2^/d and 8.5 mm^2^/d in CR, PR, SD, and PD patients, respectively. PD patients could be subclassified into a cohort with an increase in TGR (64%, PD TGR^pre-to-post-BL INCR^) and a decrease in TGR (36%, PD TGR^pre-to-post-BL DECR^) from pre- to post-BL. PD TGR^pre-to-post-BL DECR^ patients exhibited similar OS to patients classified as SD, while PD TGR^pre-to-post-BL INCR^ patients had significantly shorter OS (65 days vs 471 days, *p* < 0.001).

**Conclusions**

The use of TGR^pre-BL^ and its change to TGR^post-BL^ determined at 30-day FU1 showed better OS prognostication for patients with PD. This modification of the Lugano classification should be explored as a novel imaging biomarker of early response.

### O7 Advanced cellular characterization via modified UNET framework in high-throughput microscopy imaging

#### Polat Goktas^1,2^, Ricardo Simon Carbajo^1,2^

##### ^1^UCD School of Computer Science, Dublin, Ireland; ^2^CeADAR: Ireland’s Centre for Applied Artificial Intelligence, Dublin, Ireland

###### **Correspondence:** Polat Goktas (polat.goktas@ucd.ie)

*Cancer Imaging 2023,* **23(1):**O7

**Aim**

The objective was to develop a novel UNet binary segmentation framework to mitigate systematic noise in high-throughput microscopy imaging, which can result in false-negative results in virtual cell staining, enhancing cellular analytics accuracy.

**Materials and Methods**

Our model diverges from the standard UNet architecture, utilizing a discrete Fourier-transform module strategy and a specialised training procedure for 512 x 512 tiled images. Training involved mapping brightfield images to binarised nuclei stain targets using label-free images and nuclei stain targets from Hoechst dye measurements of bone marrow-derived human mesenchymal stem cells.

**Results**

Our model outperformed the standard UNet model in both training and testing metrics. The training loss was 0.191 and the validation loss was 0.258 compared to 0.217 and 0.308 of the standard UNet, respectively. Higher mean values of accuracy, precision, recall, and F1 scores were achieved on the test dataset (0.842, 0.862, 0.852, and 0.850, respectively) than those of the standard UNet. The modified model also effectively mitigated the impact of cross-structure artifacts on image borders, leading to enhanced segmentation accuracy.

**Conclusions**

The proposed framework can notably enhance accuracy and reproducibility of cellular analytics in large-scale image datasets, thereby facilitating high-throughput, label-free imaging studies in clinical settings. The modifications made to the network structure have enhanced segmentation outcomes and improved accuracy in capturing cellular features. This innovative approach offers potential advancements in the fields of cancer screening, diagnostics, and therapeutic imaging, thereby elevating the quality of high-throughput microscopy imaging.

### O8 The role of Ga-68 FAPI PET/CT in breast cancer treatment response assessment and follow-up

#### Yael Eshet^1,2^, Noam Tau^3,2^, Michal Eifer^1,2^, Meital Nidam^1^, Einav GalYam^1^, Liran Domachevsky^1,2^

##### ^1^Sheba Medical Center, Ramat Gan, Israel; ^2^Tel Aviv University, Tel Aviv, Israel; ^3^Sheba Medical Center, Ramat Gan, Israel

###### **Correspondence:** Yael Eshet (yael.eshet@gmail.com)

*Cancer Imaging 2023,* **23(1):**O8

**Purpose**

Ga-68 fibroblast activation protein inhibitor (FAPI), a new PET/CT radiotracer targeting cancer-associated fibroblasts in tumour microenvironment, can detect many types of cancer. We aimed to assess whether it can also be used for response assessment to treatment and follow-up.

**Materials and Methods**

We followed patients with FAPI avid invasive lobular breast cancer (ILC) before and after treatment changes, and correlated qualitative maximal intensity projection (MIP) images and quantitative tumour volume with CT results and blood tumour biomarkers.

**Results**

Six consenting ILC breast cancer patients (53 ± 8 years old) underwent a total of 24 scans (baseline for each patient and 2-4 follow-up scans). We found a strong correlation between Ga-68 FAPI tumour volume and blood biomarkers (*r* = 0.7, *p* < 0.01), but weak correlation between CT and Ga-68 FAPI MIP-based qualitative response-assessment.

**Conclusions**

We found a strong correlation between ILC progression and regression and Ga-68 FAPI tumour volume. Ga-68 FAPI PET/CT could possibly be used for disease response assessment and follow-up.

### O9 Radiologic-histopathologic registration for biological validation of prostate cancer radiomics signatures

#### Ana Sofia Castro Verde^1,2^, Ana Mascarenhas Gaivão^3^, Celso Matos^3^, Gonçalo Froes^4^, Jorge Fonseca^4,5^, Raquel Cruz Conceição^2^, Nikolaos Papanikolaou^1,6^

##### ^1^Computational Clinical Imaging Group, Champalimaud Research, Champalimaud Foundation, Lisbon, Portugal; ^2^Instituto de Biofísica e Engenharia Biomédica, Faculdade de Ciências, Universidade de Lisboa, Lisbon, Portugal; ^3^Radiology Department, Champalimaud Clinical Center, Champalimaud Foundation, Lisbon, Portugal; ^4^Urology Unit, Champalimaud Clinical Center, Champalimaud Foundation, Lisbon, Portugal; ^5^Instituto de Ciências Biomédicas Abel Salazar, Universidade do Porto, Porto, Portugal; ^6^Radiology Department, The Royal Marsden Hospital, Sutton, United Kingdom

###### **Correspondence:** Ana Sofia Castro Verde (ana.castroverde@research.fchampalimaud.org)

*Cancer Imaging 2023,* **23(1):**O9

**Aim**

Multi-parametric Magnetic Resonance Imaging (mpMRI) offers improved sensitivity to detect clinically significant prostate cancer. Radiomics allows the extraction of high-throughput tumour characteristics to permit an early diagnosis. There is a need to perform biological validation to establish a causal relationship between the extracted radiomics features and the biopsy ground-truth. In this study, we aim to address the challenging intermediate task of multimodal image registration between mpMRI and histopathology.

**Methods**

A Python script using itk-elastix was created to perform slice-by-slice registration between the histopathology slide and the corresponding T2-weighted mpMRI slice. A set of automated control points was extracted from corresponding slices using the Harris corner detector in scikit-image and a binary prostate mask was segmented on the fixed T2-weighted MRI to guide registration. After an initial alignment of the geometric centres, a similarity followed by a deformable b-spline registration was performed.

**Results**

Our registration solution was tested in a total of eighty-three slices from twenty-five prostate cancer patients from the publicly available The Cancer Imaging Archive (TCIA) PROSTATE-MRI dataset. Qualitatively we observe a good overlap between registered modalities. We have computed an average Dice similarity coefficient between the fixed prostate mask and the corresponding resulting image mask of 0.86 ± 0.06.

**Conclusion**

This work is relevant to identify biologically validated radiomics signatures for prostate cancer that could be used to improve patient stratification. Future steps include the radiologic-histopathologic correlation on a prospective cohort of one hundred radical prostatectomy prostate cancer patients undergoing mpMRI acquisitions.

### O10 Radiopsy: WBMRI-ADC quantitative features for discrimination of smoldering and multiple myeloma in a prospective study

#### Giacomo Feliciani^1^, Alice Rossi^2^, Emiliano Loi^1^, Giada Sancini^2^, Andrea Prochowski Iamurri^2^, Danila Diano^2^, Arrigo Cattabriga^2^, Matteo Marchesini^3^, Domenico Barone^2^, Anna Sarnelli^1^, Claudio Cerchione^3^

##### ^1^Medical Physics Unit, IRCCS Istituto Romagnolo per lo Studio dei Tumori "Dino Amadori", Meldola, Italy; ^2^Radiology Unit, IRCCS Istituto Romagnolo per lo Studio dei Tumori "Dino Amadori", Meldola, Italy; ^3^Translational-Hematology Unit IRCCS Istituto Romagnolo per lo Studio dei Tumori “Dino Amadori”, Meldola, Italy

###### **Correspondence:** Giacomo Feliciani (giacomo.feliciani@irst.emr.it)

*Cancer Imaging 2023,* **23(1):**O10

**Aim**

Distinguishing between Smouldering and Multiple Myeloma at staging using ADC quantitative features.

**Materials and Methods**

From 2021 to 2023, we enrolled consecutive myeloma patients into an observational prospective trial and divided them into two groups: High-risk Smouldering Multiple Myeloma and Multiple Myeloma. All patients underwent WB-MRI and an experienced radiologist and medical physicist placed four cylindrical VOIs labeled as LI, LS, RI, RS, on the pelvis bone and two on D11 and L5 vertebrae. Quantitative features were extracted from the ROIs using S-IBEX, an IBSI-compliant software. LASSO algorithm was used to build logistic regression models, which were then validated using the test set. Receiver operating curves (ROC) and area under the curve (AUC) were used as metrics for models’ performance assessment.

**Results**

The study included 80 patients with 45 diagnosed with MM and 35 with SMM. 144 quantitative features were extracted from the 6 VOIs contoured on WB-MRI ADC sequence for each patient. After applying ICC and LASSO algorithm, 21 stable features were obtained, and logistic regression models showed a median AUC of 0.85 (0.79-0.95) in the training phase and a median AUC of 0.65 (0.55-0.80) in the test phase. The best predictive model had an AUC of 0.95 and 0.75 in the training and test phase, respectively. Notably, the strongest predictor for distinguishing between MM and SMM was the ADC mean value for each VOI location.

**Conclusion**

Stable radiomics features extracted from six VOIs contoured on ADC sequences can distinguish between MM and SMM with good performance.

### O11 Treatment response evaluation by PET/CT and CT after neoadjuvant chemotherapy in advanced ovarian cancer

#### Elaine YP Lee, Ka-Yu Tse, Philip PC Ip

##### University of Hong Kong, Hong Kong, Hong Kong

###### **Correspondence:** Elaine YP Lee (eyplee77@hku.hk)

*Cancer Imaging 2023,* **23(1):**O11

**Aim**

Imaging is commonly used to evaluate the treatment response to neoadjuvant chemotherapy (NACT) in advanced ovarian cancer (OC). However, the additional value of PET/CT over contrast-enhanced CT (ceCT) in unclear in this setting. Aim of the study was to compare the diagnostic accuracies of PET/CT and ceCT after NACT.

**Methods**

Patients with newly diagnosed stage III-IV OC scheduled for NACT were prospectively recruited. PET combined with ceCT was performed after 3-6 cycles of NACT before interval debulking surgery. Abdominopelvic cavity was divided into 19 regions and scored for the presence of residual disease. Intra-operative findings or histology were taken as standard of reference. Diagnostic characteristics described by accuracy (Acc), sensitivity (Sen), specificity (Spe), positive predictive value (PPV) and negative predictive value (NPV) of PET and ceCT were compared using McNemar’s test.

**Results**

Forty-two patients (median 55, 41-76-years-old) were recruited with 793 regions available for analysis. PET had an overall Acc 87%, Sen 49%, Spe 97%, PPV 83% and NPV 88%; ceCT had an overall Acc 88%, Sen 64%, Spe 94%, PPV 75% and NPV 91%. No significant difference was demonstrated between the two imaging modalities (p>0.05). Right subdiaphragmatic and bowel serosa disease were commonly missed by both modalities and PET also missed residual omental disease due to resolution of the metabolic uptake after NACT.

**Conclusion**

PET had no additional value over ceCT with comparable diagnostic accuracy in detecting residual disease after NACT in advanced OC, commonly missing disease at the right subdiaphragmatic space and bowel serosa.

### O12 Quantification of visceral and subcutaneous fat on CT as predictors of outcome in endometrial cancer

#### S Jain, S Chandola, X Li, S Ghaem-Maghami, A Rockall

##### Department of Surgery and Cancer, Imperial College London, London, United Kingdom

###### **Correspondence:** S Jain (shrujal.jain19@imperial.ac.uk)

*Cancer Imaging 2023,* **23(1):**O12

**Aim**

To assess the association of visceral obesity (determined on CT) with tumour characteristics and survival outcomes in an endometrial cancer (EC) cohort and a subgroup of stage 1 disease.

**Methods**

Patients included in this study were reviewed at Hammersmith Hospital between 2012 and 2018. The visceral fat area (VFA) and subcutaneous fat area (SFA) were segmented on preoperative CT scans at the L3 vertebral level. The VFA to SFA ratio (VSR) was then calculated for 318 patients (220 of which had stage 1 disease). The full SFA was not visible for the remaining 26 patients. For survival analysis, VSR cut-offs that produced maximal survival difference were identified and Kaplan-Meier (with log-rank test) or cox proportional hazards models were used.

**Results**

Endometrioid EC was associated with larger VFA than non-endometrioid EC (*p* = 0.004). Age was the only characteristic associated with VSR (Spearman’s correlation coefficient=0.239, *p* < 0.001). The median and interquartile range of follow-up for death and progression were 7.0 years (6.0–8.1 years) and 4.4 years (1.0–5.7 years) respectively. VSR was not predictive of disease-specific or overall survival in the whole cohort or stage 1 (all *p* > 0.05). However, high VSR was associated with significantly lower progression-free survival (PFS) probability than low VSR in the whole cohort and stage 1 (*p* = 0.046 and *p* = 0.017 respectively). On multivariate analysis, VSR was only an independent predictor of PFS in stage 1 (hazard ratio 2.29, 95% confidence interval 1.05–5.50, *p* = 0.038).

**Conclusion**

VSR is a potential prognostic marker of PFS in stage 1 EC.

## Posters

### P1 Intra-abdominal haemorrhage from spontaneous rupture of Visceral tumours: CT Findings

#### Valerio Arpaia^1^, Maria Iovino^2^, Francesca Rosa Setola^3^, Valeria Macchia^3^, Emilio Vicenzo^3^, Luca Sanduzzi^1^, Salvatore Maione^3^, Fabio Sandomenico^3^

##### ^1^Diagnostic Imaging and Radiotherapy Departement, Azienda Ospealiera Universitaria Federico II, Naples, Italy; ^2^San Giuliano Hospital, Giugliano (Naples), Italy; ^3^Radiology Unit, Buon Consiglio Fatebenefratelli Hospital, Naples, Italy

###### **Correspondence:** Fabio Sandomenico (f.sandomenico@virgilio.it)

*Cancer Imaging 2023,* **23(1):**P1

**Learning Objectives**

To recognise key imaging findings from Contrast-Enhanced Computed Tomography (CECT) of emergency situations due to intra-abdominal haemorrhage from spontaneous rupture of visceral tumours.

**Content Organisation**

Spontaneous intra-abdominal haemorrhage is the abnormal presence of blood into the abdominal cavity in absence of traumas. It causes either haemoperitoneum or haemoretroperitoneum depending on haemorrhagic site. Although non-traumatic intra-abdominal bleedings are globally rare, spontaneous tumour rupture is one of the most frequent noxae. Clinical presentation is typically non-specific, and a conclusive diagnosis should always be made with imaging findings. In the setting of emergency, multi-phase Contrast-Enhanced Computed Tomography (CECT) is the procedure of choice due to its low temporal resolution and its capability to detect even small amounts of intra-abdominal haemorrhage. In this paper we illustrate the main anatomical and clinical features of visceral tumours that are more prone to spontaneous rupture (hepatocarcinoma, hepatic adenoma and haemoangioma, adrenal tumours, kidney tumours, splenic sarcomas, etc.). Then, we describe key CT findings of spontaneous tumour rupture such as presence of a clot in the haemorrhagic site (sentinel clot sign), contrast extravasation and time-dependent density change of haematoma in different CT phases.

**Conclusion**

CECT scanning is a pivotal technique for intra-abdominal haemorrhage from spontaneous visceral tumour rupture. In these situations, independently from tumour type, it displays an almost unique series of findings to achieve either a conclusive diagnosis or a correct therapeutical management.

### P2 CT assessment of advanced ovarian cancer: findings precluding primary cytoreductive surgery

#### Stephan Edey, Anum Pervez, Olwen Westerland, Audrey Jacques, Sultana Hasso, Savithri Rajkumar, Rahul Nath, Gautem Mehra, Ahmad Sayasneh, Sarah Natas

##### Guy's and St. Thomas' Hospital, London, United Kingdom

###### **Correspondence:** Stephan Edey (sarahnts.6@gmail.com)

*Cancer Imaging 2023*, **23(1):**P2

**Learning objectives**

Advanced ovarian cancer: demonstration of specific sites of disease on CT imaging which preclude primary debulking surgery, in the light of new ultraradical surgical techniques.

**Content organisation**

Primary cytoreductive surgery (debulking) is the treatment of choice in advanced ovarian cancer, followed by adjuvant chemotherapy.

Improved survival is associated with optimal debulking with the aim of as little residual disease as possible. This has led to newer aggressive ultraradical surgical techniques; however, despite this, certain sites of disease may still be unresectable. These should be recognised preoperatively to ensure patients do not undergo inappropriate potentially morbid surgery, with the alternative to downstage the disease with neoadjuvant chemotherapy.

This pictorial review will show examples of key sites of disease spread which preclude primary debulking surgery. We will focus on CT which still forms the majority of preoperative imaging staging.

Sites include:

- Infiltrative mesenteric root disease
- Confluent porta hepatis and coeliac axis disease
- Diffuse small bowel serosal disease or extensive segmental sites of bowel involvement
- Multi site hepatic parenchymal disease
- Invasion or encasement of large vessels (IVS/aorta/iliac)
- Multi-site and confluent thoracic disease

**Conclusions**

Despite new ultra radical cytoreductive surgery for patients with advanced ovarian cancer, certain sites of disease remain unresectable. It is important to recognise these to avoid inappropriate surgery.

### P3 Abdominal emergencies in oncologic patients: "Elementary, my dear Watson"

#### Eleni Tsakirmpaloglou^1^, Georgia Mingou^1^, Anatoli Stoforiadi^2^, Reggina Goulimari^2^, Melanie Sahinidou^1^, Olga Nikolaidou^1^

##### ^1^Radiology Department, General Hospital, "G. Papanikolaou", Thessaloniki, Greece; ^2^Radiology Department, General Hospital of Xanthi, Xanthi, Greece

###### **Correspondence:** Olga Nikolaidou (olganikolaidou@hotmail.com)

*Cancer Imaging 2023*, **23(1):**P3

**Learning Objectives**

- To introduce the prevalence of abdominal complications in oncologic patients.
- To depict the commonest abdominal entities leading to the emergency department.
- To highlight the importance of imaging and especially computed tomography in the detection of oncology emergencies.
- To illustrate the imaging features of primary or secondary complications derived from abdominal malignancies.

**Content organisation**

Oncologic emergencies consist of any acute and potentially life-threatening events, derived from the direct effects of the underlying condition or as a post-therapy complication. Approximately, 40% of cancer patients present to the emergency department with gastrointestinal symptoms.

Oncologic emergencies can be categorised as metabolic, haematologic, and structural based. The latter remain of paramount importance to be recognised and pointed out by the radiologists, as imaging remains the standard method of choice in detecting such conditions. Abdominal emergencies are usually derived from the primary tumour. Nevertheless, in patients with advanced cancer stage, III or IV, may be an indirect consequence related to its systemic manifestations.

The gold standard and modality of choice in the evaluation of cancer patients is Contrast-enhanced computed tomography (CT), providing important findings in major abdominal complications. Thus, CT multiplanar reconstruction offers additional information in structural based complications.

We will point out the imaging characteristics of acute abdominal oncologic conditions involving the gastrointestinal and the hepatobiliary tract, the vascular system and lastly the urinary tract.

**Conclusion**

Oncologic emergencies are not exceptional and may be life-threatening. Therefore, they demand paramount awareness to get identified and to establish the correct diagnosis, promptly.

###  P4 Deep invasion volume of primary nasopharyngeal carcinoma is a strong predictor of outcome

#### Qi Yong H Ai^1,2^, Ann D. King^2^, Ho Sang Leung^2^, Lun M. Wong^2^, Frankie K.F. Mo^2^

##### ^1^The Hong Kong Polytechnic University, Kowloon, Hong Kong; ^2^The Chinese University of Hong Kong, Shatin, Hong Kong

###### **Correspondence:** Qi Yong H Ai (hemis.ai@polyu.edu.hk)

*Cancer Imaging 2023,* **23(1):**P4

**Aim**

The primary tumour volume of nasopharyngeal carcinoma (NPC) shows only a weak association with outcome and little impact on T-staging. However, the volume of deep tumour invasion, especially as a ratio to the superficial component, may be a marker of aggressiveness and predictor of outcome.

**Materials and Methods**

The MRI of 743 patients with NPC were retrospectively evaluated. Three primary tumour volume measurements were obtained, total volume (Vtotal), deep invasion volume (Vdeep) and ratio of deep to total volume (Vratio). Volumes were correlated with 5-year disease-free survival (DFS) using cox regression with step-forward approach to select the optimal V-related predictor. The optimal V-related predictor, together with other confounding factors (T, N, overall stage, sex, age and treatment) were then added into the multivariate model to identify the independent predictors for predicting DFS. Hazard ratio (HR) was calculated. A *p* < 0.05 indicated statistically significant.

**Results**

Vratio outperformed the other two volume measurements for predicting DFS (HR = 3.56, *p* <0.01), a higher ratio had poorer outcome. The multivariate analysis showed that Vratio (HR = 3.18) and N stage (HR = 1.94) (*p* < 0.01) were the only two independent predictors for DFS in the multivariate analysis.

**Conclusion**

Primary NPCs that show greater volume of deep invasion relative to the superficial component have a poorer outcome and this ratio outperforms Vtotal and T-staging.

### P5 Dual energy CT applications in oncologic imaging

#### Nils Grosse Hokamp, Thorsten Persigehl

##### University Hospital and University of Cologne, Cologne, Germany

###### **Correspondence:** Nils Grosse Hokamp (nils.grosse-hokamp@uk-koeln.de)

*Cancer Imaging 2023,* **23(1):**P5

**Objective**

The objective of this educational exhibit is to provide an overview of dual-energy CT (DECT) and its applications in oncologic imaging. It aims to highlight the advantages of DECT in improving tissue characterisation, functional analysis, and reducing metal artifacts.

**Content Organisation**

This abstract is organised into three sections. The first section introduces technology and reconstructions from DECT and sets the stage for discussing applications in oncologic imaging.

The second section discusses how DECT enables improved tissue characterisation by differentiating between tumour tissue, healthy tissue, and necrotic or haemorrhagic areas. It also explores the functional information provided by DECT through the generation of iodine maps, facilitating the assessment of tumour vascularity and treatment response. Furthermore, it emphasises DECT's ability to reduce metal artifacts, which is particularly relevant in oncology patients with metallic implants or prior surgeries.

The final section concludes the abstract by summarising the key points. It underscores the value of DECT in oncologic imaging, highlighting its potential to improve diagnostic accuracy, treatment planning, and monitoring of oncology patients. It also emphasises the importance of continued advancements in DECT technology and glimpses at further innovation enabled by photon counting CT.

**Conclusion**

Dual-energy CT is a powerful tool in oncologic imaging that offers numerous advantages. Its ability to improve tissue characterisation, provide functional information through iodine mapping, and reduce metal artifacts makes it an invaluable resource for oncology patients. In light of increasing availability of DECT reconstructions, knowledge of fundamental concepts and common pitfalls is beneficial.

### P6 Chemotherapy-Related Cognitive Impairment (CRCI) in breast cancer: an ALE meta-analysis of neuroimaging studies

#### Sonya Utecht, Linda Larson-Prior

##### University of Arkansas for Medical Science, Little Rock, USA

###### **Correspondence:** Sonya Utecht (sutecht@uams.edu)

*Cancer Imaging 2023*, **23(1):**P6

**Aim**

Worldwide over 1.8 million cases of breast cancer are diagnosed per year, with critical treatments leading, in many cases, to chronic cognitive dysfunction in affected individuals. Chemotherapy-related cognitive impairment (CRCI) or “chemo-brain” is a disruptive side effect of adjuvant chemotherapy plaguing many breast cancer survivors. To better understand the effects on brain network organisation, several neuroimaging studies have examined individuals suffering from CRCI. However, it can be difficult to draw conclusions from these studies as many have small sample sizes, disagreements on anatomic terminology, and varied foci. The goal of this study is to perform a meta-analysis of these studies to provide a clearer understanding of those functional brain networks underlying the cognitive dysfunction in breast cancer patients.

**Methods**

Using a PRISMA framework, search queries were performed using PubMed and Google Scholar with a variety of terms including “neuroimaging”, “CRCI”, “functional connectivity”, “chemotherapy", and “breast”. After filtering, 43 potential studies were identified, 7 of which had relevant image coordinate data and were entered into analysis in our pilot study. Activation Likelihood Estimation meta-analyses were performed using the GingerALE toolkit.

**Results**

Coordinate data from these studies were fed into the GingerALE software system. The hippocampus, amygdala, and bilateral insula showed significant differences between the 184 chemotherapy-positive breast cancer survivors and the 132 chemotherapy-naive controls.

**Conclusion**

The resulting meta-analyses provide evidence of decreased connectivity in regions associated with the default mode network, as well as increased connectivity in hippocampal regions associated with reduced neuropsychological test results and memory function.

### P7 Influence of the “Streamline Phenomenon” on the laterality of hepatic metastases from colorectal cancer

#### Eduardo De Araujo, Eva Rolim, Rubens Chojniak

##### Ac Camargo Cancer Center, Sao Paulo, Brazil

###### **Correspondence:** Eduardo De Araujo (eduardo.mparaujo@gmail.com)

*Cancer Imaging 2023*, **23(1):**P7

**Aim**

To discuss the concepts behind the theory of streamline flow in the distribution of liver metastases depending on the origin of the primary lesion in the colon and the impact of this phenomenon on liver metastatic recurrence-free survival in patients undergoing liver metastasectomy.

**Materials and Methods**

Retrospective study, in patients with colorectal neoplasia (adenocarcinoma) from 2016 to 2023. A total of 144 patients in the study who were divided into two groups according to the origin of the colon lesion side, right (45) and left (99) by counting the number of metastases in each hepatic lobe by sectional imaging methods. Follow-up of 58 patients to assess liver recurrence after colectomy and liver surgery according to surgical laterality (ipsilateral, contralateral and both lobes).

**Results**

The mean number of metastases in the right hepatic lobe was 5.22 (right colon) and 4.50 (left colon); and mean of 2.22 in the left lobe (right colon) and 3.42 (left colon), without significant differences, but with numerical differences (OR= 19) when in the right colon. There was no difference in the liver recurrence curve regardless of surgical laterality [(14) ipsilateral; (11) contralateral; (33) both lobes].

**Conclusions**

There was no significant difference in the laterality of liver metastasis. However, it was observed that isolated metastases tend to follow the laterality of the primary lesion, especially when the primary lesion is on the right.

**Fig. 1 (abstract P7).**
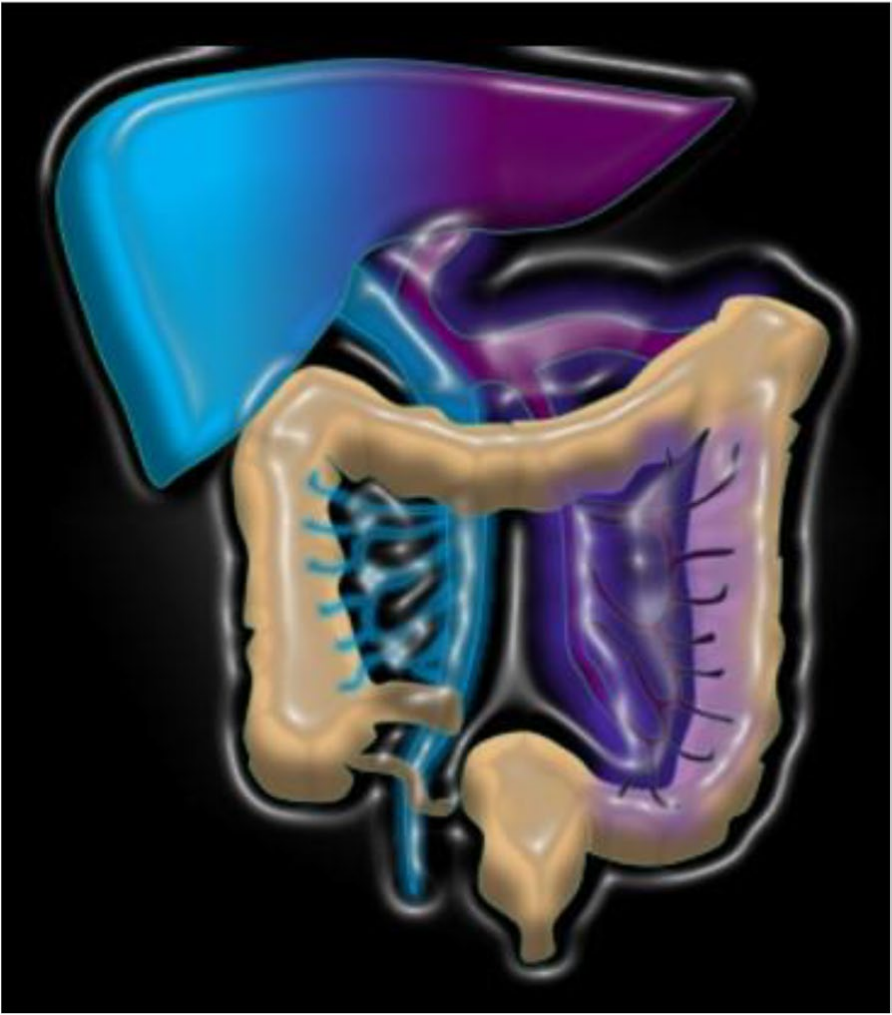
See text for description

### P8 F-18 FDG, F-18 PSMA, and Cu-64 DOTATATE PET/CT: One patient, three cancers

#### Ryan Rahman, Fathima Palot Manzil, Joshua Eichhorn

##### University of Arkansas for Medical Sciences, Little Rock, USA

###### **Correspondence:** Ryan Rahman (jmeichhorn13@gmail.com)

*Cancer Imaging 2023*, **23(1):**P8

**Learning Objectives**

To review the impact of appropriate PET radiotracer selection in identification and differentiation of malignancy and metastatic disease based on the patient’s primary tumour type.

**Content Organisation**

Selection of radiotracers is fundamental to the proper evaluation and diagnosis of malignancy, whether primary or metastatic. The choice of radiotracer is often chosen based on a combination of cancer history, suspicion for certain cancer types, and the indication for PET/CT – whether for surveillance or staging purposes.

We will discuss three well-known radiotracers in PET/CT imaging: F-18 FDG, F-18 PSMA, and Cu-64 DOTATATE; this will include:

- A brief overview of the mechanism of each radiotracer uptake and imaging protocol
- Indications for specific radiotracer selection
- Advantages and disadvantages of each radiotracer

We will discuss a case utilising all three of these radiotracers in a single patient with multiple primary malignancies to highlight:

- Real-life selection and application of these aforementioned PET radiotracers based on clinical suspicion in conjunction with patient history
- The importance of follow-up imaging after false-negatives in PET surveillance

**Conclusion**

F-18 FDG, F-18 PSMA, and Cu-64 DOTATATE radiotracers are common tools in the radiologist’s arsenal for visualisation of primary malignancy and metastatic disease on PET/CT scans. Knowing the underlying mechanism, in accordance with benefits and limitations of each tracer, allows for patient-specific selection. This can be especially crucial when there is suspicion for multiple underlying primary malignancies.

### P9 AI for prostate MRI: results from a large multi-centre, multi-vendor external validation study

#### Aarti Shah^1^, Nadia Moreira da Silva^2^, Michael Yeung^2^, Francesco Giganti^3^, Lucy Davies^2^, Paul Burn^4^, Richard Hindley^5^, Nikhil Vasdev^6,7^, John Hayes^6^, Sophie Squire^1^, Alison Bradley^8^, Giles Maskell^8^, Adrian Andreou^9^, Sidath Liyanage^10^, Mark De Bono^10^, Raj Persad^11^, Jon Aning^11^, Nimalan Sanmugalingam^12^, Tristan Barrett^12,13^, Mark Hinton^2^, Antony Rix^2^, Evis Sala^14^

##### ^1^Hampshire Hospitals NHS Foundation Trust, Winchester, United Kingdom; ^2^Lucida Medical Ltd, Cambridge, United Kingdom; ^3^University College London, London, United Kingdom; ^4^Somerset NHS Foundation Trust, Taunton, United Kingdom; ^5^University of Winchester, Winchester, United Kingdom; ^6^East and North Herts NHS Trust, Stevenage, United Kingdom; ^7^University of Hertfordshire, Stevenage, United Kingdom; ^8^Royal Cornwall Hospitals NHS Trust, Truro, United Kingdom. ^9^Royal United Hospitals Bath NHS Foundation Trust, Bath, United Kingdom; ^10^Mid and South Essex NHS Foundation Trust, Southend, United Kingdom. ^11^North Bristol NHS Trust, Bristol, United Kingdom; ^12^Cambridge University Hospitals NHS Foundation Trust, Cambridge, United Kingdom; ^13^University of Cambridge, Cambridge, United Kingdom; ^14^Policlinico Universitario A. Gemelli, IRCCS, Dept. of Radiology, Rome, Italy

###### **Correspondence:** Aarti Shah (Aarti.Shah@hhft.nhs.uk)

*Cancer Imaging 2023*, **23(1):**P9

**Aim**

Evaluate how an AI decision support system for MRI in prostate cancer generalises to multi-centre datasets including multiple scanner models, vendors, field strengths and imaging protocols.

**Methods** AI-based software for analysis of prostate MRI was developed using data from PROSTATEx and five sites in a diagnostic study (794 patients, 34% csPCa). It was subsequently evaluated on blinded external validation data (mpMRI, 252 patients, 31% csPCa) from six sites, including one unseen site/scanner. Exclusions included prior treatment and image quality issues. The software automatically outputs scores intended to identify Gleason score (GS)≥3+4 csPCa. csPCa was confirmed by biopsy (GS≥3+4/PI-RADS ≥3), with PI-RADS 1/2 patients with no biopsy assumed negative. Performance was evaluated using ROC analysis, with 95% confidence intervals estimated by bootstrapping.

**Results**

At the pre-determined threshold, AI identified patients with csPCa with sensitivity 94% (95% CI 88-99%), specificity 57% (49-64%), and NPV 95% (90-99%) on the blinded external validation dataset. AUC was 0.85 (0.80-0.90). Per-site AUC ranged from 0.70-0.98, with pooled AUC 0.86±0.11.

Reporting radiologists had per-patient sensitivity 99% (95% CI 96-100%) due to the assumed ground truth, specificity 73% (67-80%), NPV 99% (98-100%), and AUC 0.95 (0.92-0.97) on this dataset. In a 2019 Cochrane meta-analysis of 12 major studies (37% csPCa), radiologists identified patients with GS≥3+4 csPCa with sensitivity 86% and specificity 42%.

**Conclusion**

In a large external validation, this AI system shows comparable performance to radiologists in major expert studies, indicating promising potential for AI to support PCa detection in clinical practice.

### P10 Integrating clinical data with AI to optimise decision-making in prostate MRI

#### Antony Rix^1^, Nadia Moreira da Silva^1^, Jobie Budd^1^, Michael Yeung^1^, Francesco Giganti^2^, Lucy Davies^1^, Paul Burn^3^, Richard Hindley^4,5^, Nikhil Vasdev^6,7^, Alison Bradley^8^, Giles Maskell^8^, Adrian Andreou^9^, Sidath Liyanage^10^, Raj Persad^11^, Jon Aning^11^, Tristan Barrett^12,13^, Mark Hinton^1^, Anwar Padhani^14^, Evis Sala^15^

##### ^1^Lucida Medical Ltd, Cambridge, United Kingdom; ^2^University College London, London, United Kingdom; ^3^Somerset NHS Foundation Trust, Taunton, United Kingdom; ^4^Hampshire Hospitals NHS Foundation Trust, Winchester, United Kingdom; ^5^University of Winchester, Winchester, United Kingdom; ^6^East and North Herts NHS Trust, Stevenage, United Kingdom; ^7^University of Hertfordshire, Stevenage, United Kingdom; ^8^Royal Cornwall Hospitals NHS Trust, Truro, United Kingdom; ^9^Royal United Hospitals Bath NHS Foundation Trust, Bath, United Kingdom; ^10^Mid and South Essex NHS Foundation Trust, Southend, United Kingdom; ^11^North Bristol NHS Trust, Bristol, United Kingdom; ^12^Cambridge University Hospitals NHS Foundation Trust, Cambridge, United Kingdom; ^13^University of Cambridge, Cambridge, United Kingdom; ^14^Paul Strickland Scanner Centre, Northwood, United Kingdom; ^15^Policlinico Universitario A. Gemelli, IRCCS, Dept. of Radiology, Rome, Italy

###### **Correspondence:** Antony Rix (Aarti.Shah@hhft.nhs.uk)

*Cancer Imaging 2023*, **23(1):**P10

**Aim**

Evaluate accuracy of multi-modal decision support models for prostate cancer combining imaging AI, clinical data and PI-RADS scores.

**Methods**

MRI, clinical history, histopathology and PI-RADS data were obtained retrospectively from a five-site, multi-vendor, multi-scanner study. 352 patients were used for training, and 235 patients (Gleason grade group (GGG)≥2, prevalence 34%) for held-out test. GGG≥2 was used as ground truth with MRI-negative patients assumed negative. Multi-modal models combining scores from separately-developed multi-stage MRI analysis AI software, clinical data, and original PI-RADS scores were trained. Sensitivity, specificity and AUC were evaluated at patient level on held-out data, and compared to AI-score and PI-RADS assessment alone.

**Results**

PI-RADS scores identified patients with GGG≥2 with sensitivity 1.00 (95% CI 1.00-1.00), specificity 0.67 (0.61-0.75) and AUC 0.94 (0.91-0.97).

AI detected patients with GGG≥2 with sensitivity 0.97 (0.93-1.00), specificity 0.55 (0.47-0.62) and AUC 0.88 (0.84-0.92), using bpMRI data.

Combining AI score and TZ-PSA density (PSAD) gave sensitivity 0.95 (0.90-0.99, *p* < 0.001), specificity 0.70 (0.63-0.77, *p* < 0.001) and AUC 0.90 (0.85-0.93, *p* = 0.25).

Combining AI, PSAD and PI-RADS gave sensitivity 0.99 (0.96-1.00, *p* < 0.001), specificity 0.83 (0.77-0.89, *p* < 0.001) and AUC to 0.96 (0.93-0.98, *p* = 0.003).

TZ volume based PSAD had modest additional benefit compared to whole-prostate PSAD. Other variables offered <5% specificity improvements or non-significant benefits. Findings with bpMRI and mpMRI AI models were similar.

**Conclusion**

Combining PI-RADS, PSAD and AI scores offers considerable specificity improvement, at similar sensitivity, compared to AI or PI-RADS assessments alone. This could substantially benefit selection of patients for biopsy using MRI.

### P11 Lung carcinoma with atrial invasion

#### JH Wong, Z Haron

##### Institut Kanser Negara, Putrajaya, Malaysia

###### **Correspondence:** JH Wong (jhueyw@gmail.com)

*Cancer Imaging 2023,* **23(1):**P11

**Learning objectives**

To review the radiological signs of atrial invasion and pathways of spread by lung carcinoma on computed tomography (CT).

**Content organisation**

Cardiac invasion by lung carcinoma is a rare occurrence, however, potentially life-threatening. The predisposition for cardiac involvement depends on the histological subtype, tumour location and stage of disease.

We will present the imaging features of atrial invasion by lung carcinomas, including

- the characteristics indicative of atrial invasion
- its differentiation from atrial thrombus and benign cardiac masses

We will review the pathways of tumour spread such as lymphatic spread, haematogenous spread, direct invasion and transvenous extension.

We will also discuss the potential limitations of CT in diagnosing atrial invasion.

**Conclusions**

Recognition of radiological findings on CT of atrial invasion by lung cancers is important as awareness of this rare entity is essential for timely diagnosis and appropriate treatment planning in patients with lung carcinoma.

### P12 Radiomics for the differential diagnosis between ameloblastomas and odontogenic keratocysts on panoramic radiography

#### Kuo Feng Hung^1^, Qi Yong H Ai^2^

##### ^1^Applied Oral Sciences & Community Dental Care, Faculty of Dentistry, The University of Hong Kong, Hong Kong, Hong Kong; ^2^The Hong Kong Polytechnic University, Hong Kong, Hong Kong

###### **Correspondence:** Kuo Feng Hung (hungkfg@hku.hk)

*Cancer Imaging 2023*, **23(1):**P12

**Aim**

To compare the performance of radiomics models built with and without an ensemble technique in discriminating between ameloblastomas and odontogenic keratocysts (OKCs) on panoramic radiography (PR).

**Methods**

86 pre-operative PRs with ameloblastomas (*n* = 31) or OKCs (*n* = 55) were retrospectively included. Radiomics features were extracted from labelled lesion regions, and the dataset was split into training and testing sets (7:3 ratio). Z-score normalisation was applied to all features. For the ensemble technique, a five-fold cross-validation was conducted in the training set to select the best-performing classifier among seven machine learning classifiers. During each fold, feature selection was performed using t-tests (*p* < 0.05) and LASSO, and each classifier was trained using the selected features. An ensemble approach was utilised to aggregate the best-performing classifier trained corresponding to the five-fold cross-validation through their weighted sum. The performance of the ensemble model was tested on the testing set using AUC and their 95% CIs. For the non-ensemble technique, the classifier identified as the best-performing classifier was trained using features selected by t-tests and LASSO. Hyperparameter tuning was performed using grid search and five-fold cross-validation. The model’s performance was assessed on the testing set.

**Results**

Among seven classifiers, the ensemble support vector machine (SVM) outperformed others in discriminating between ameloblastomas and OKCs on PR. The ensemble model achieved an AUC of 0.89 (95%CI 0.74-0.99) while the non-ensemble SVM achieved an AUC of 0.67 (95%CI 0.44-0.89).

**Conclusion**

The ensemble model's performance in discriminating between ameloblastomas and OKCs on PR was more reliable than the non-ensemble SVM.

### P13 The role of alternate diagnosing imaging in the era of LI-RADS for diagnosing hepatocellular carcinoma

#### WHK Chiu^1^, BCK Chow^1^, JC Ng^2^, KDD Leung^1^, WTV Chan^3^

##### ^1^Queen Elizabeth Hospital, Hong Kong, Hong Kong; Kwong Wah Hospital, Hong Kong, Hong Kong; ^3^Tuen Mun Hospital, Hong Kong, Hong Kong

###### **Correspondence:** WHK Chiu (keith.chiu@ha.org.hk)

*Cancer Imaging 2023,* **23(1):**P13

**Aim**

The Liver Imaging Reporting and Data System (LI-RADS) has improved and standardised reporting of liver lesions. Unlike other guidelines, the use of more than one imaging modality to achieve non-invasive diagnosis of Hepatocellular Carcinoma (HCC) appears limited. This study aims to examine whether combining Computed Tomography (CT) and Magnetic Resonance Imaging (MRI) improves the performance of LI-RADS in HCC detection.

**Materials and Methods**

Between 2020 and 2022, consecutive patients at risk for HCC who underwent CT and MRI within 4 months of each other for liver lesion characterisation were retrospectively analyzed. Two radiologists rated all treatment-naive lesions in consensus using LI-RADS v2018. Inter-modality agreement and diagnostic performance were evaluated using composite outcomes as ground truths.

**Results**

In total, 66 patients (median age 62 [IQR 58-70years], 26 [39.4%] female) with 115 lesions (median SLD 1.2, IQR 0.8-1.8cm, 17 [14.8%] HCC) were included. LI-RADS major features showed good inter-modality agreement (rho=0.74, *p* < 0.0001) and ancillary features changed LI-RADS classifications in 18 (15.6%) lesions. The highest combined LI-RADS classification has an accuracy of 91.3% when LR4/5 were considered to be HCC. Using two imaging modalities significantly improve sensitivity compared to CT alone (82.4% vs 41.2%, *p* = 0.03) without compromising specificity (92.9% vs 97.9%, *p* = ns). No significant improvement in sensitivity nor specificity was seen compared to MRI alone (76.5% and 94.9%, *p* = ns).

**Conclusion**

For indeterminate liver lesions on CT, performing MRI could improve HCC detection and avoid diagnostic delays while no such benefit is seen if the lesion was indeterminate on MRI.

### P14 Value of metabolic tumour volume as a risk stratificator for supplementing staging in NSCLC

#### Alexander Brose^1,2^, Jochem König^3^, Gabriele Anja Krombach^2^, Mathias Schreckenberger^4^, Jutta Kappes^5^, Matthias Miederer^1,4^

##### ^1^Department of Translational Imaging in Oncology, National Center for Tumour Diseases (NCT/UCC) Dresden, Germany: German Cancer Research Center (DKFZ), Heidelberg, Faculty of Medicine and University Hospital Carl Gustav Carus, University of Technology Dresden (TUD), Helmholtz-Zentrum Dresden-Rossendorf (HZDR), Dresden, Germany; Department of Diagnostic and Interventional Radiology, University Hospital Giessen, Giessen, Germany; ^3^Institute of Medical Biostatistics, Epidemiology and Informatics (IMBEI), University Medical Center Mainz, Johannes Gutenberg-University Mainz, Mainz, Germany; Department of Nuclear Medicine, University Medical Center Mainz, Johannes Gutenberg-University Mainz, Mainz, Germany; Department of Internal Medicine/ Pulmonary Medicine, Catholic Hospital Koblenz-Montabaur, Koblenz, Germany

###### **Correspondence:** Alexander Brose (abrose312@gmail.com)

*Cancer Imaging 2023*, **23(1):**P14

**Aim**

Whole-body metabolic tumour volume (MTVwb) has proven to be an independent prognostic value for non-small cell lung cancer (NSCLC) in randomised controlled trials. We hypothesised that its implementation could also be beneficial in routine clinical use to supplement staging of NSCLC.

**Methods**

We performed a single-centre retrospective analysis on 251 patients with histologically proven NSCLC who underwent FDG-PET/CT for initial staging in the years 2017 to 2019. Two readers determined baseline-MTVwb as suggested by the PERCIST criteria in a semiautomatic fashion. Agreement between readers was measured by intraclass correlation coefficient (ICC). Cox regression and Kaplan-Meier survival analyses were performed to determine the prognostic value of UICC stage and MTVwb.

**Results**

Median overall survival (OS) was 40 ± 4.7 months (95% CI 30.8 – 49.4). Agreement between the two readers for MTVwb was almost perfect (ICC 0.983). Both, UICC stage and MTVwb were independent prognostic parameters in the cox regression analyses (*p* < 0.001). When stratified into risk groups by dividing the MTVwb into quartiles (5.4, 24.4 and 109.0 mL), survival analysis showed that higher MTVwb was associated with worse OS, equally to the corresponding UICC stage. When stratified by UICC stage, higher MTVwb still showed worse overall survival in locally advanced disease and metastatic disease (*p* < 0.05).

**Conclusion**

MTVwb as suggested by PERCIST is easy to reproduce and its prognostic value is comparable to the UICC stages. Combined with the TNM staging system, it could be used for further improvement of pretreatment assessment in patients with locally advanced and metastatic NSCLC.

### P15 MRI surveillance of liver metastasis in high-risk uveal melanoma

#### Chloe McMurray, Sarah MacPherson

##### NHS Greater Glasgow and Clyde, Glasgow, United Kingdom

###### **Correspondence:** Chloe McMurray (chloe.mcmurray4@nhs.scot)

*Cancer Imaging 2023,* **23(1):**P15

**Aim**

Up to 50% of patients with uveal melanoma develop metastatic disease, most frequently in the liver (77-94%). Surveillance methods vary across centres. A consensus statement was released for metastatic surveillance in Scotland in 2019 – high risk patients are recommended to have baseline MRI liver with contrast, followed by 6-monthly MRI liver without contrast.

This study aimed to identify compliance with consensus statement guidelines on liver metastasis surveillance for high-risk uveal melanoma patients.

**Materials and Methods**

A Radiology Information System (RIS) search using key words identified patients with high-risk uveal melanoma over a 10-year period (2012-2022) who had liver MRI. Retrospective analyses were performed of the use of MRI with and without contrast before and after the introduction of the consensus statement. Caldicott and local RIS approval was granted.

**Results**

Prior to the consensus statement, 29.5% had MRI for metastatic surveillance, all of which had contrast. 30% of these were performed due to suboptimal views with ultrasound.

16 high risk cases were identified following the consensus statement. 19% had surveillance as per the recommendation. 69% had MRI follow-up, however, these were performed with contrast. Of this cohort, 12% developed liver metastases.

### P16 Morphological and functional parameters of MRI in PET/CT-proven local pancreatic cancer recurrences

#### Dusan Saponjski^1,2^, Aleksandra Djuric Stefanovic^1,2^

##### ^1^University Clinical center of Serbia, Belgrade, Serbia; ^2^Faculty of Medicine, University of Belgrade, Belgrade, Serbia

###### **Correspondence:** Email: saponjski.d@gmail.com

*Cancer Imaging 2023,* **23(1):**P16

Introduction

Prognosis of pancreatic adenocarcinoma is very unfavourable. Only 15-20% of carcinoma is operable at the time of diagnosis. During postoperative follow-up, recurrent tumours are found in 36-63% of patients, approximately 11 months after surgery.

Material & Methods

Abdominal MRI examination was performed according to the standard protocol, the existence of local recurrence was noted, as well as morphological (localisation and size), and functional parameters in the form of ADC values (diffusion coefficient). PET/CT measured maximal standardised values of radiopharmaceutical uptake (SUVmax), as well as size and localisation of the lesions. MRI and PET/CT findings were compared and correlated using Wilcoxon's equivalent pair test (Z) and Sperman's (rS) correlation coefficient.

Objective

To analyse whether MRI findings agree and correlate with PET/CT as a reference standard.

Results

23 patients were included in study and elevated serum tumour marker values (CA19-9) were found in 16 (70%). Ten patients (43.5%) had local recurrence between SMA and SMV, and another 10 patients had local recurrence around PV in the hepatoduodenal ligament. Only 2 patients had local recurrence in the projection of anastomosis and 1 had a "muff" around SMA. Average ADC value was 1.49x10-3 mm2/s. Average SUVmax was 7.8g/ml. Maximal recurrence diameter measured on MRI and PET/CT did not differ significantly (*p* = 0.375). A negative correlation was found between lesion size and ADC values (*p* = 0.005) and a positive correlation between size and SUVmax (*p* < 0.001).

Conclusion

MRI is a reliable method in detecting local pancreatic cancer recurrence, using morphological and functional parameters.

### P17 Colocolic intussusception caused by multiple grouped Vanek’s tumours: a case report

#### Dusan Saponjski

##### University Clinical Center of Serbia, Belgrade, Serbia. Faculty of Medicine, University of Belgrade, Serbia

###### **Correspondence:** Dusan Saponjski (saponjski.d@gmail.com)

*Cancer Imaging 2023,* **23(1):**P17

**Introduction** Inflammatory fibroid polyp (IFP) is a benign polypoid tumour arising from the submucosa of the gastrointestinal tract. It is most commonly located in the gastric antrum, small bowel and rarely in the colon and oesophagus. Clinically it presents with abdominal pain and gastrointestinal bleeding, but in certain cases bowel intussusception or obstruction can be seen. The condition is often discovered incidentally on imaging or endoscopy. Treatments include surgery or endoscopic resection. Herein we report a very rare case of colocolic intussusception, in a middle-aged male, caused by multiple IFP.

**Case outline**

A 63-year-old male patient was admitted to our clinic as an emergency with abdominal pain, loss of appetite and absence of stool and gas for the last two days. On imaging studies (abdominal ultrasound and computed tomography) colocolic intussusception in the right iliac fossa was observed. Patient underwent emergency surgery with right hemicolectomy. After making full recovery patient was discharged on the sixth postoperative day.

**Conclusion**

Inflammatory fibroid polyps (IFPs) are a rare cause of colic intussusception and as such cannot be definitively differentiated from intussusceptions caused by malignant lesions; therefore radical surgery is often advised.

Consent to publish had been obtained from the patient.

### P18 Developing a day-case Radiologically-inserted Gastrostomy (RIG) service

#### Rishi Chavda, Neel Raja, Steven Jepson, Bhavini Billimoria

##### University Hospitals of Leicester NHS Trust, Leicester, United Kingdom

###### **Correspondence:** Neel Raja (neelraja@doctors.org.uk)

*Cancer Imaging 2023,* **23(1):**P18

**Aim**

RIG catheters are an invaluable adjunct in providing nutritional support for patients unable to tolerate oral intake and in whom percutaneous endoscopic gastrostomy is not a viable option. Within our centre, all patients undergoing RIG insertion were admitted, with inpatient stays of up to seven days. Our study aimed to determine if procedures could be carried out safely as a day-case. Barriers to transitioning to day-case were explored, of which post procedure analgesia requirements and complications of insertion were acknowledged.

**Materials and Methods**

A pilot study including ASA grade I or II patients requiring RIG insertion was performed. Data was collected retrospectively utilising electronic patient records. Post procedure analgesia requirements for day-case procedures were analysed, ensuring adequate analgesia on discharge. Follow up data was collected on complication rates including mortality, major morbidity and readmission for pain.

**Results**

18 day-case RIG procedures were performed in total with 100% technical success. There were no immediate or delayed complications, nor readmissions for pain. One patient was re-admitted with a minor complication of cellulitis around the RIG site 18 days post-procedure. The 30-day all-cause mortality rate was 0.

**Conclusions**

Our pilot study regarding the feasibility of performing day-case RIG procedures in a carefully selected patient cohort has demonstrated clear safety and economic advantages with successful pre-procedural planning of discharge analgesia. Given this, the service will be continued and shared with neighbouring trusts.

### P19 Sorting the imaging: improved metadata indices of the cancer imaging archive

#### Tracy Nolan, Michael Rutherford, Kirk Smith, Lawrence Tarbox

##### University of Arkansas for Medical Sciences, Little Rock, USA

###### **Correspondence:** Tracy Nolan (tnolan@uams.edu)

*Cancer Imaging 2023,* **23(1):**P19

**Aim**

The Cancer Imaging Archive (TCIA) is an NCI-supported open-science repository of projects called Collections, serving access to 40TB of medical imaging data to 10,000 users across 100 nations each month. Improving access to TCIA metadata is vital as the sophistication of medical imaging interpretation via informatics techniques evolves.

The purpose of this project was to survey and index Collection-level imaging-supporting details as part of an infrastructure migration project.

**Method**

Each TCIA Collection landing page holds imaging and metadata; however understanding a Collection through its metadata requires traversing multiple interfaces and interpreting unstructured content.

A survey of 200 Collection pages (by June 2023) was conducted using manual (browser-based interface, GUI) and programmatic interface (API) review of summaries, imaging files, detailed descriptions, linked research articles and external content. This survey was tabulated to index the presence of details including:

- Inclusion/Exclusion/Treatment description (20%)
- Image processing provenance (40%)
- Code (18% with links)
- Funding acknowledgment (48%)

Results

Labels at the Collection level for these indices are then applied to Collection pages serving data through GUI and API. This approach will continue to enhance TCIA, with the potential to be applied retrospectively.

Conclusion

Enhancing the data abstraction used for searches, addresses the cancer imaging community's growing demand for more refined comparisons within TCIA. These advanced search filters both:

- assist TCIA's users in constructing new cohorts to download from existing TCIA Collections
- provide opportunities to structure contextual metadata during TCIA’s users upload and curation of new TCIA Collections.

### P20 Boosting cancer therapy: a supervised clustering based on interpretable machine learning optimised mesenchymal cell separation

#### Polat Goktas^1,2^, Ricardo Simon Carbajo^1,2^

##### ^1^UCD School of Computer Science, Dublin, Ireland; ^2^CeADAR: Ireland’s Centre for Applied Artificial Intelligence, Dublin, Ireland

###### **Correspondence:** Polat Goktas (polat.goktas@ucd.ie)

*Cancer Imaging 2023,* **23(1):**P20

**Aim**

We aimed to improve the characterisation and separation of Mesenchymal Stem Cells (MSCs) used in cancer cell therapies through an efficient, interpretable machine learning approach. This method differentiates MSCs under varying serum conditions, a crucial factor influencing their therapeutic efficacy.

**Materials and Methods**

Human MSCs derived from bone marrow were cultivated under healthy (serum-containing) and stressed (low-serum containing) conditions, reflecting varied environments in cancer treatment. These cells were then analyzed through Bright-field (BF) images using a tree-based machine learning model framework. We employed a 2-D discrete Fourier (DFT) module and used Shapley Adaptive eXplanations (SHAP) values in a supervised clustering approach, eliminating the need for potentially damaging chemical staining.

**Results**

The DFT module application increased the accuracy of the Random Forest model from 80.15% to 93.26%, enhancing our ability to differentiate MSCs in diverse serum conditions, especially reducing false-negative results. We effectively identified MSC characteristics on label-free images by transforming raw data into SHAP values. Accordingly, we employed a supervised clustering method to convert the raw data into SHAP values using Random Forests, enhancing the differentiation and separation of MSCs on these label-free images.

**Conclusions**

Our machine learning methodology offers a significant step forward for real-time, automated characterisation of MSCs, crucial in enhancing the precision and effectiveness of cell-based cancer therapies. This label-free assessment approach promises to greatly enhance the viability and effectiveness of cell-based therapies.

### P21 Reliability analysis of a previously proposed MRI radiomics model for the detection of nasopharyngeal carcinoma

#### Lun M Wong^1^, Qi-Yong Ai^2,1^, Ann D King^1^

##### ^1^The Chinese University of Hong Kong, Shatin, NT, Hong Kong; ^2^The Hong Kong Polytechnic University, Hung Hom, KL, Hong Kong

###### **Correspondence:** Lun M Wong (lun.m.wong@cuhk.edu.hk)

*Cancer Imaging 2023,* **23(1):**P21

**Background**

Despite well-known challenges in reliability of radiomics analysis, current studies often prioritise exploring new radiomics applications rather than investigating the reliability of existing radiomics signatures in the literature. In this study, we aimed to validate the reliability of an MRI radiomics signature we previously proposed and trained to discriminate between nasopharyngeal carcinoma (NPC) and benign hyperplasia in an expanded dataset.

**Methods**

An ensemble radiomics model previously trained on a dataset consisting of 221 early-stage T1 NPC patients and 221 benign hyperplasia patients was applied in this study to an detect NPC inan additional validation dataset, comprising 284 and 163 patients with NPC of all stages and benign hyperplasia, respectively. An automatic segmentation algorithm for nasopharynx lesion we previously developed was used to generate the lesion segmentation for the ensemble model. The performance was evaluated using receiver operator characteristics (ROC) analysis.

**Results**

The previously proposed model attained better areas under curves (AUCs) of 0.91 (95%CI:0.87-0.93) in the additional validation data when compared to that in the original study of 0.85 (95% confidence interval [CI]:0.82–0.89).

**Conclusion**

In conclusion, the previously proposed and trained MRI radiomics showed promising reproducibility and reliability with better performance in a new set of data in terms of AUC, which could be attributed to the addition of all NPC stages rather than just T1 NPCs that are most difficult to discriminate from benign hyperplasia.

### P22 Facing a dilemma: leiomyosarcoma or leiomyoma?

#### Antonella Kovic, Miguel Nazar, Emilia Martinez, Enzo Casali, Silvina De Luca

##### Hospital Alemán, CABA, Argentina

###### **Correspondence:** Antonella Kovic (sdeluca@hospitalaleman.com)

*Cancer Imaging 2023,* **23(1):**P22

**Learning objectives**

Harnessing the potential of MRI in addition to morphological features on nonenhanced postcontrast sequences, DW imaging and ADC measurement may have a potential ability to differentiate uterine sarcomas from benign leiomyomas.

**Content organisation**

Leiomyomas are the most common benign smooth muscle tumours of the uterus, they occur in perimenopausal as well as reproductive-age woman. On the opposite side, leiomyosarcomas are the malignant spectrum, leiomyosarcomas are the most common uterine sarcomas.

Leiomyosarcomas and leiomyomas share a similar clinical presentation as rapidly growing uterine tumours.

MRI is widely accepted as the standard for preoperative evaluation, leiomyosarcomas commonly presenting as large uterine masses with haemorrhage and necrosis, with high signal on T1-weighted and T2-weighted images and the presence of unenhanced pocket-like areas on contrast-enhanced sequences.

Sarcomas and cellular leiomyomas show high signal intensity on DWI images, whereas ordinary leiomyomas and most degenerated leiomyomas showed low signal intensity. The mean ADC value of sarcomas was 1.17 +/- 0.15, which was lower than those of the normal myometrium (1.62 +/- 0.11) and degenerated leiomyomas (1.70 +/- 0.11).

**Conclusion**

It is crucial to be able to differentiate between leiomyomas and leiomyosarcomas on imaging, as therapeutic management is different.

### P23 MRI assessment for bladder cancer: unveiling the VI-RADS classification

#### Antonella Kovic, Miguel Nazar, Emilia Martinez, Silvina De Luca

##### Hospital Alemán, CABA, Argentina

###### **Correspondence:** Antonella Kovic (sdeluca@hospitalaleman.com)

*Cancer Imaging 2023,* **23(1):**P23

**Learning objectives**

To point out the importance of this entity to show the role of MRI sequences in the diagnosis and local staging of bladder cancer.

**Content organisation**

Up to 90% of bladder tumours are urothelial carcinomas. Urothelial tumours are classified as either invading muscle (non papillary) or not invading muscle (superficial or papillary).

Urothelial carcinomas of the bladder take two divergent patterns of tumourigenesis with very different prognostic significance and long-term survival. They are classified as either non-muscle-invasive or muscle-invasive. The assessment of muscle invasion is essential in the staging and treatment of bladder cancer: it drives the surgical approach with either resection with or without bacillus Calmette-Guerin instillations or radical cystectomy with or without neoadjuvant chemotherapy or immunotherapy.

The bladder wall contains four defined layers: the urothelium, the highly vascular lamina propia (submucosa), the muscularis propia and the outermost serosa.

VI-RADS score, derived using T2-weighted MRI, diffusion-weighted imaging, and dynamic contrast enhancement, which suggests the risks of muscle invasion.

VI-RADS score consisting of five categories describing the likelihood of muscle invasion. Scores 1 and 2 are assigned to tumours unlikely to invade the muscularis propria, whereas scores 4 and 5 are assigned to Bladder Cancer likely to infiltrate the detrusor muscle layer

**Conclusions**

MRI imaging technology have made multiparametric MR imaging a feasible and reasonably accurate technique for the local staging of bladder to optimise treatment.

### P24 Role of 18 FDG PET CT in oesophageal cancer

#### Emilia Martinez, Antonella Kovic, Silvina De Luca, Cecilia Carrera

##### Hospital Alemán, CABA, Argentina

###### **Correspondence:** Emilia Martinez (sdeluca@hospitalaleman.com)

*Cancer Imaging 2023,* **23(1):**P24

**Learning objectives**

To review the value of 18 FDG PET CT in the staging, tumoural response assessment and detection of recurrence in oesophageal cancer.

**Content organisation**

Oesophageal cancer is a major cause of morbidity and mortality worldwide. Obtaining accurate pre-treatment and follow up evaluation, allows stage-appropriate treatments, optimising oesophageal cancer outcomes.

The expanding therapeutic options available for treating patients in this condition demands precise staging. Various methods are currently employed to stage oesophageal cancer. However, no single modality can accurately stage every patient on its own, making necessary the use of a combination of modalities.

An accurate and thorough methodology in the staging process is indispensable to classify different patients, providing a treatment that presents the highest likelihood of cure and the lowest risk of morbidity.

We will discuss the role of 18 FDG PET CT in the management of oesophageal cancer:

- Benefits and restrictions of this integrated method
- Other non-invasive and invasive methods, as a complement to PET-CT.

We will present clinical cases of our hospital, with different oesophageal cancer stages and outcomes:

- Stages according to the 8th edition of American Joint Committee on Cancer.
- Favourable and unfavourable response to neoadjuvant treatment and surgery.
- Recurrence of the disease.

Conclusions

18 FDG PET CT plays an essential role in the staging and restaging of oesophageal cancer, providing a detailed description of the primary tumour invasion of paraoesophageal structures, lymph node involvement and distant metastases, all in one single method.

### P25 Reducing the carbon footprint of cancer imaging storage

#### YiFan Jia^1^, Rebecca Burger^2^, Michael Deng^2^, Andrea Rockall^2^

##### ^1^Imperial College London, London, United Kingdom; ^2^Imperial College Healthcare NHS Trust, London, United Kingdom

###### **Correspondence:** YiFan Jia (jyf0811@gmail.com)

*Cancer Imaging 2023,* **23(1):**P25

**Aim**

The increasing burden of imaging storage in PACS contributes significantly to Radiology’s carbon footprint. CT studies are used extensively in cancer imaging. Modern PACS allows common reformats to be generated instantaneously from the original thin slice image in CT studies. However, multiple reformats and duplicates are often also permanently stored, increasing carbon footprint without clinical benefit. This study aims to evaluate the carbon emission associated with this additional storage.

**Methods**

183 initial staging CT studies of endometrial cancer patients between 2013-2018 were retrospectively analysed. The number of reformats (multiplanar reformats, maximum intensity projections images, lung reconstructions) were recorded for each study. The file size of each reformat was recorded for the first 30 of these studies.

Scenario analyses of carbon emissions were performed based on literature review, the Imperial College Healthcare Trust endometrial cancer scanning protocol, and projected incidence statistics by Cancer Research UK.

**Results**

97% of studies had reformats and/or duplicates. Coronal reformats were the most common (91%), followed by sagittal reformats and lung reconstructions (85%). The original image had a mean size of 290mb, averaging 36% of the total file size. Of the reformats, sagittal had the largest mean size of 219mb.

Removing all reformats and duplicates from long-term storage would save an estimated 53566 kgCO2e (or 45951 kgCO2e if only lung reconstructions were kept) over the next 20 years. This equates to powering 270 homes over this period.

**Conclusion**

Updating CT storage protocols can significantly reduce imaging associated carbon footprint without impeding clinical interpretation.

### P26 Pseudocirrhosis post-chemotherapy breast cancer metastatic liver, our experience

#### Cecilia Carrera, Cintia Barale, Silvina De Luca, Antonella Kovic, Eugenia Costa, Paula Insaurralde

##### Hospital Alemán, CABA, Argentina

###### **Correspondence:** Silvina De Luca (sdeluca@hospitalaleman.com)

*Cancer Imaging 2023,* **23(1):**P26

**Learning objectives**

- Describe radiologic findings in pseudocirrhosis to differentiate it from oncologic disease progression and to be aware of adequate treatment.
- Illustrate imaging and pathology correlation.

Content organisation

Breast cancer is one of the most prevalent malignancies in our environment and worldwide, oncologic control through CT will allow us to detect the response to chemotherapy and the appearance of undesirable effects such as pseudocirrhosis.

Pseudocirrhosis is a radiologic term that describes the hepatic effects which occur in patients with primary or secondary tumours treated with chemotherapy drugs, especially from breast cancer. It is expressed as capsular retraction, irregularity and nodularity of the liver contour, atrophy of the right lobe, hypertrophy of the left lobe and caudate with signs of portal hypertension, of rapid progression. The changes produced are similar to macronodular liver cirrhosis secondary to chronic liver disease and are differentiated by histopathology.

Conclusion

Chemotherapy can be hepatotoxic, conditioning the appearance of entities such as pseudocirrhosis in metastatic breast cancer with hepatic involvement, increasing comorbidity. Knowledge for the radiologist is crucial allowing an early diagnosis.

### P27 Outcomes of targeted liver biopsy in patients presenting acutely with metastatic cancer

#### M J Khalid, M Tan, M Mahadi, S Muthukumarasamy

##### Hull University Teaching Hospitals NHS Trust, Hull, United Kingdom

###### **Correspondence:** S Muthukumarasamy (siva.muthukumarasamy@nhs.net)

*Cancer Imaging 2023,* **23(1):**P27

**Aim**

Ultrasound-guided liver biopsy referral can be made in patients with metastatic cancer during an acute presentation. In this study we review outcomes of procedure and patient survival post-biopsy. Any subsequent oncological intervention was also recorded.

**Materials and Methods**

We retrospectively analysed 57 patients at our tertiary care hospital over an 18-month period. Patient demographics, laboratory markers, disease burden, histology, procedural success, complications and oncological interventions were analysed.

**Results**

Median age at presentation was 71.5 years (47-90).

Overall median survival was 49.5 days (mean 88 days). 30-day mortality was seen in 23 patients (40%), death within 1 to 3 months was seen in 18 patients (31.5 %), and 10 patients (17.5%) died within 3 to 6 months post-biopsy with only 2 patients (3.5 %) surviving beyond 12 months. Patients with 30-day mortality were seen to have >10 liver metastasis in 20 cases (median survival 15.5 days) and 4 cases had concurrent >10 liver and lung metastasis (median survival 17 days).

Patients with CRP >180 mg/L on presentation had poor outcome.

Biopsy was successful in 54 patients (95%), and post-biopsy haemorrhage was encountered in 2 patients (3.5%) requiring embolisation.

Only 12 patients (21%) had specialist multi-disciplinary (MDT) input before biopsy and overall, 23 patients (40%) proceeded to have oncological intervention post-biopsy.

**Conclusions**

Metastatic cancer has poor 12-month survival in emergency presentation. Disease burden, performance status, other prognostic indicators in line with NICE guidelines, should be considered when making decisions about liver biopsy in these patients.

### P28 Comparison of ADC and PSA density for risk stratification of MRI Likert 3 prostate lesions

#### Paul Burn^1^, Mathuri Sakthithasan^1^, Neil Trent^1^, Nick Burns-Cox^1^, Dow-Mu Koh^2^

##### ^1^Musgrove Park Hospital, Taunton, United Kingdom; ^2^Royal Marsden Hospital, London, United Kingdom

###### **Correspondence:** Paul Burn (Paul.Burn@somersetft.nhs.uk)

*Cancer Imaging 2023,* **23(1):**P28

**Aim**

To compare ADC and PSAD for the assessment of MRI Likert 3 prostate lesions.

**Methods**

Consecutive patients, previously investigated for prostate cancer with 3T MRI (Siemens Vida) and biopsy, with a Likert 3 score on MRI report, were retrospectively reviewed. Contemporaneous Gleason group (GG) and PSA were recorded. We measured ADC (x 10-3 mm2/s) of the index lesion on MRI by placing an ROI through its centre. For ADC and PSAD respectively, the medians of two groups, no significant cancer (benign and GG1) (NSC) and significant cancer (≥GG2) (SC) were compared (Mann-Whitney U test). We assessed the diagnostic performance of ADC and PSAD to identify significant cancers by ROC analysis.

**Results**

108 patients included, mean age 65 years (48-82 years), histology: 62 benign, 25 GG1, 17 GG2, 4 GG4/5.

The median ADC (IQR) of NSC group 1.1 (0.9-1.3) was higher than the SC group 0.89 (0.7-1.0) [*p* < .001]. There was no significant difference in the median PSAD (IQR) between NSC group 0.10 (0.06-0.16) versus SC group 0.13 (0.09-0.17) (*p* = 0.0783).

The diagnostic accuracy (AUC) of using ADC to determine the presence of significant cancer was 0.798 (0.698-0.899). A threshold of <1.1 for significant cancer showed NPV 95% (95% CIs 85-99%), sensitivity 86% (64-97%), specificity 60% (49-70%), PPV 34% (23-48%).

**Conclusion**

ADC is superior to PSAD for differentiating patients with and those without significant cancer in the context of MRI Likert 3 score, and may provide adjunct information to support biopsy decision-making in this patient group.

### P29 Review of sentinel node SPECTCT for nodal staging in cutaneous melanoma

#### Mustafa Al-Alawi, Michael Kay, Rachel Plant

##### University Hospitals Dorset, Poole, United Kingdom

###### **Correspondence:** Michael Kay (michael.kay@uhd.nhs.uk)

*Cancer Imaging 2023,* **23(1):**P29

**Abstract**

With the introduction and increasing use of adjuvant treatment for cutaneous melanoma over the past 5 years, the use of sentinel node SPECTCT in initial staging has considerably increased.

At University Hospitals Dorset we have more than doubled the number of sentinel node imaging studies, performing 81 in the year to August 2022, with further increases over the recent period.

We present an audit of the technical aspects, including failure rate and postulated causes, of the service for all studies performed August 2021-2022 and discuss the adaptations we have made in terms of patient bookings and technetium MAA dosing in order to adapt to the increasing volume of referrals.

Finally, we discuss the impact on patient care including the improved prognostic conversations that can be had with patients due to the accurate nodal staging afforded by sentinel node biopsy and BRAF status. All potential sentinel node cases are discussed in the regional MDT between dermatology, surgery and oncology and we outline the discussion steps and considerations undertaken for each patient.

### P30 Radiology pathway navigation – a new direction

#### Louisa Edwards-Brown, Sarah Maund

##### Princess of Wales Hospital, Bridgend, United Kingdom

###### **Correspondence:** Louisa Edwards-Brown (louisa.edwards@wales.nhs.uk)

*Cancer Imaging 2023*, **23(1):**P30

**Aim**

The patient journey through diagnosis and treatment for suspected cancer is complex, with multiple investigations, the pathway process can be upsetting and confusing for patients causing unnecessary anxiety. Streamlining Radiology pathways would provide more clinical coordination for patients with connections between Radiology and clinical teams allowing more prompt diagnosis and optimal treatment options. Implementation of a new Advanced Practice role - Radiology Cancer Navigator - was required to achieve the changes and enable collaborative working.

**Methods**

A Radiology Navigator has provided a more synchronous patient journey - attending MDTs/Cancer Manager Meetings, a point of contact for patients and clinical teams, undertaking referral vetting to reduce delays, managing referrals, coordinating multiple examinations, fulfilling own clinical caseload, and improving patient information including digital access.

**Results**

- Reduced vetting procedure for USC referrals to 1 day
- New staging pathway for positive colonoscopy patients, down to 4 days (National optimal pathway within 10 days)
- GP chest x-ray to staging CT down - 10 to 7 days
- Increased capacity – extra 555 patients scanned
- Radiologist time saving 6hrs 40mins per week, Managers/Radiology staff – 12hrs 30mins per week
- Staff feedback – 96% of staff felt they had seen improvements in or experienced less obstacles in first 6 months of Navigator role.

**Conclusion**

An innovative change which streamlined and reduced delays in Radiology pathways. A positive addition of an Advanced Practice Radiography role bringing benefits to patients and staff.

